# Diarrheal diseases prevalence among children of Sudan and socio cultural risks related; systematic review and meta analysis

**DOI:** 10.1186/s12879-023-08920-7

**Published:** 2024-01-02

**Authors:** MM Badawi, MA SalahEldin, AB Idris, EB Idris, SG Mohamed

**Affiliations:** 1 Higher Academy for Strategic and Security Studies, Khartoum, Sudan; 2 Medical Microbiology Department, Faculty of Medical Laboratory Sciences, University of Khartoum, Khartoum, Sudan; 3 General Surgery Resident, Sudan Medical Specialization Board, Khartoum, Sudan; 4 Department of Medical Microbiology, Rashid Medical Complex, Riyadh, Saudi Arabia

**Keywords:** Developing countries, Gastritis, Africa

## Abstract

It is estimated that more than 4% of the causes of inpatient deaths in 2017 were due to diarrheal diseases. The current study is aimed to provide pooled prevalence of microorganisms causing diarrhea among Sudanese as well as determine any socio-cultural risk factors associated. A systematic review of the literature was conducted and regulated in accordance to PRISMA. After abstract and full text screening Twenty-one research articles were recruited to the study. Among witch eighteen research articles determined prevalence of causative microorganisms of Diarrhea; eight research articles determining prevalence of causative microorganisms of Diarrhea were conducted in Khartoum State, five in Gezira State, two in White Nile State, one in Kordofan State while two studies were conducted in several States. Moreover, majority of studies were concerned of prevalence among children while two studies were toward general population as well as mothers of children. The pooled prevalence of viral diarrhea in less than five years old children was 22.90% [15.37, 30.43] among more than 14 thousands’ participants, the pooled prevalence of parasitic diarrhea was 31.40% [19.53, 43.27] among participants from different age groups while the pooled prevalence of bacterial diarrhea was 36.20% [14.00, 58.40]. No associated risk factors were able to be synthesized from included studies.

**Supplementary Information:**

The online version contains supplementary material available at 10.1186/s12879-023-08920-7.

## Introduction

Bearing in mind the political issues that have weighed down Sudan with war and enmity for the last 30 years, health care has become an afterthought and basically lost in the midst of what governments might believe to be more pressing matters. The country faces escalating humanitarian devastation, almost 8 million people facing critical problems related to mental and physical wellbeing, including more than 1.5 million internally displaced people and almost 1 million refugees. Resources are scarce, economic output is shrunken by two-thirds between 2017 and 2018 and the country's health system is unprepared to respond to growing and neglected needs. Adding insult to injury, Sudan still has a long way to go to achieve the Sustainable Development Goals (SDGs). According to the WHO as well as the Sudan Health Observatory in the federal ministry of health, the major communicable diseases contributing (SHO) to morbidity are Malaria, Tubercelosis, Schistosomiasis, Pneumonia and Diarrheal diseases [1, 2].

Diarrheal diseases and despite of the encouraging trends in the positive direction regarding water quality and good sanitation nationally in Sudan, diarrheal deaths attributable to lack of tolerable water, sanitation as well as hygiene remain a considerable challenge. In 2015, 26% of people in Sudan were reported to practice open defecation and a further 30% relied on unimproved forms of sanitation, while access to basic and safely managed drinking water was reported to be variable; ranging from 95% in Khartoum State down to 30% in North Darfur State. SHO estimated that more than 4% of the causes of inpatient deaths in 2017 were due to diarrheal diseases, specific determination causative pathogen prevalence determination is rarely conducted in Sudan as even reference hospitals in several localities are not equipped to detect and identify several microorganisms causing diarrhea [1, 2]. For research purposes; several studies investigated fecal specimens collected from children with diarrhea in Sudan; they determined varying estimates ranged from 2% up to 20% for different viral pathogens (*rotavirus, norovirus, adenovirus, bocavirus*), a range of 1% up to 15% for different bacterial pathogens (*Shigella, E.coli, Vibrio spp, and Salmonella spp*) and a range of 16% up to 35% for different parasitic pathogens [3–8].

Knowledge of causative agents that cause diarrheal disease is critical in the implementation of suitable actions to prevent and control these diseases. The current study is aimed to provide pooled prevalence of microorganisms causing diarrhea among Sudanese as well as to determine any socio-cultural risk factors associated.

## Materials and methods

### Search strategy

To identify relevant studies; a systematic review of the literature was conducted in the 1st of June 2022 as described in details previously [9]. The review was regulated in accordance with the PRISMA (Preferred Reporting Items for Systematic Reviews and Meta-Analyses) Statement [10] (S1 Table). A comprehensive search was operated in PubMed, Embase, Google scholar, Scopus, Index Copernicus, DOAJ, EBSCO-CINAHL, Cochrane databases without language limits. To obtain a current situation evidence; only studies published in or after 2010 were included. Furthermore, all studies where the data collection process took place before 2010 were also excluded, the only exception was if the collection process started in or before 2010 and ended in 2010 or afterwards.

As medical literature in Sudan is generally scarce in international databases as well as socio-cultural factors may be differently reported, socio-cultural factors were not used in keyword formulation and their related results were later extracted from included studies, the keywords used in PubMed was as follow:

Diarrhea[Mesh] OR Gastroenteritis[Mesh] OR gastrointestinal infection[Mesh] OR enteritis[Mesh] OR dysentery[Mesh]AND Sudan*[tiab]. As previously described [11].

Moreover, to optimize our search, hand searches of reference lists of included articles were also performed.

### Study selection and data extraction

Titles and abstracts were assessed for preliminary eligibility. A copy of the full text was obtained for all research articles that were available and approved in principle to be included. Abstraction of data was in accordance with the task separation method; method and result sections in each study were separately abstracted in different occasions to reduce bias. Moreover, data abstraction was conducted with no consideration of author’s qualifications or expertise. Each research article was screened for all relevant information and recorded in the data extraction file (Microsoft Excel), data from each method section was extracted using a predefined set of variables; study characteristics, type of participants, study population size, geographical region, methodology used in prevalence or risk assessment and the period of the study conduction as described in details previously [9].

After inclusion, studies were further classified into studies determining prevalence, studies determining socio-cultural risk factors and studies determining both prevalence and socio-cultural risk factors. Furthermore, as risk factors-related keywords were not formulated in the search strategy, each study was fully screened to check the nature of the risk investigated by authors, studies determining risk factors in which socio-cultural risks have not been assessed, were later excluded.

Although age grouping is available alongside their corresponding included study in (Table 1), it was not possible to be included in the Meta analysis due to the complexity and diversity of categorization of “age” variable among studies included.

**Table 1:** Characteristics of included studies

Study ID & Year of publication	Study design	State	study population/s	Assessment of	Sample Size	Gender	Participants' Age
**Abdalla, 2013** [12]	Cross sectional	Khartoum	Suspected Children	Prevalence of Rotavirus (ELISA)	92	Both	< 1—4 years
**Adam, 2018** [13]	Cross sectional	Khartoum	Suspected Children	Prevalence of several pathogens (Nuclic acid)	437	Both	< 1—5 years
**Ahmed, 2015** [14]	Cross sectional	Khartoum	Suspected Children	Prevalence of Rotavirus (ELISA)	100	Both	< 1—< 4 years
**Algahtani & Elhassan, 2020** [15]	Cross sectional	Gezira	Mothers of children under 5 years	Risk factors	150	Both	25—45 years
**Ali, 2015** [16]	Cross sectional	Gezira	Suspected Children	Prevalence of Rotavirus (ELISA)	673	Both	< 1—< 5 years
**Eisa, 2019** [17]	Cross sectional	North Kordofan	Suspected Children	Prevalence of several pathogens (Parasitological methods)	100	Both	5—12 years
**Elmanssury, 2022** [18]	Cross sectional	Khartoum	Mothers of children under 5 years	Prevalence /risk factors	311	Both	Not determined
**Hassan, 2020** [19]	Cross sectional	Khartoum	Primary school adolescents	Prevalence of several pathogens (Parasitological methods)	134	Both	Jun-14
**Hussein, 2018** [20]	Cross sectional	Khartoum	Suspected Children	Risk factors	162	Both	< 1—< 5 years
**Ibrahim, 2015** [21]	Retrospective	Gezira	Suspected Children	Prevalence of Rotavirus (ELISA)	389	Both	< 1—< 5 years
**Imam & Osman, 2014** [22]	Cross sectional	Gezira	Children and adolescents	Prevalence of several pathogens (Parasitological methods)	400	Both	5—16 years
**Khogali, 2013** [23]	Cross sectional	Khartoum	Children and adults	Prevalence of several pathogens (Bacteriological methods)	900	Not determined	Not determined
**Magzoub, 2013** [24]	Cross sectional	Different States^a^	Suspected Children	Prevalence of Rotavirus (ELISA & Nuclic acid)	755	Both	< 1—< 5 years
**Mohamed, 2019** [25]	Cross sectional	Gezira	Suspected Children	Prevalence of Norovirus (Nuclic acid)	50	Not determined	< 1—< 5 years
**Mohamed, 2018** [26]	Cross sectional	Gezira	Suspected Children	Prevalence of Rotavirus (Nuclic acid)	66	Both	< 1—< 5 years
**Mohammed, 2018** [27]	Cross sectional	White Nile	General population	Prevalence of several pathogens (Parasitological methods & ELISA)	279	Not determined	4—85 years
**Mustafa, 2014** [28]	Cross sectional	Khartoum, Gezira, Northern State, West Darfur, Gadarif, Red sea & North Kordofan	Suspected Children	Prevalence of Rotavirus (ELISA)	10,953	Both	< 1—5 years
**Netsereab & Xenos,2017** [29]	Secondary analysis	All 18 States of Sudan	Suspected Children	Risk factors	14,081	Both	< 1—5 years
**Saeed, 2015** [30]	Cross sectional	Khartoum	Suspected Children	Prevalence of several pathogens (Bacteriological methods & Nuclic acid)	437	Both	< 1—5 years
**Subahi, 2017** [31]	Cross sectional	Khartoum	Suspected Children	Prevalence of Rotavirus (ELISA)	180	Both	< 1—> 2 years
**Tamomh,2021** [32]	Cross sectional	White Nile	Suspected Children	Prevalence of Cryptosporidium (Parasitological methods)	150	Both	< 1—5 years

^a^Further details are unavailable

### Assessment of quality

Each included article was evaluated based on a framework for making a summary assessment of the quality. The related published literature was crossed, then a framework was structured specifically to determine the level of representativeness of the studied population and to judge the strength of the estimates provided. Five questions were to be answered in each article, each answer represent either 1 score for yes, 0 score for No or 0 score for not available; a total score for risk of bias and quality was calculated by adding up the scores in all five domains, resulting in a score of between 0 and 5. The highest score indicates the highest quality, only studies with a score for quality greater or equal to 3 (higher quality) were included. The five domains were: is the study objective clearly defined?, is the study sample completely determined?, is the study population clearly defined and specified?, is the methodology rigorous? And is the data analysis rigorous?, as described in details previously [9].

### Secondary analysis

Among all included studies reporting either prevalence or risk factor estimates, articles were crossed whether Standard Error (SE) is reported. In studies where the *SE* is not reported; the following formula was used to calculate it: *SE* = √p (1-p)/ n, where *p* stands for Prevalence as described in details previously [9]. Regarding risk factors, as each included study may have different objective influencing thereby their result demonstration (i.e. adjusted OR, unadjusted OR, frequencies), each individual category in a given socio-cultural variable investigated the Odd Ratio (OR) was calculated (whenever possible) to provide univariate analysis for the given category among investigated population.

### Quantitative analysis

Meta-analysis was performed—whenever possible using Review Manager Software (Version 5.3). The software automatically provided the Confidence Interval (CI) according to the calculated *SE*, if the *CI* is provided in a study; it was introduced accordingly. The heterogeneity of each meta-analysis was assessed as well, the random effect was favored over the fixed effect model in all meta-analysis established as variations between studies is predicted to be probable due to the diversity of the study populations. Sensitivity analysis was also approached to determine the effect of studies conducted in populations proposed to behave in indifference manners or proposed to have low risk on the overall pooled data. Moreover, subgroup analysis was also conducted -whenever suitable to determine prevalence of specific pathogen or risk level in specific State or population. An outcome to take part in the meta-analysis has to be included in at least two studies as described in details previously [9].

Trim and Fill method was used to assess the risk of publication bias in each Meta analysis conducted [33].

## Results

### Studies included

**Fig. 1:**
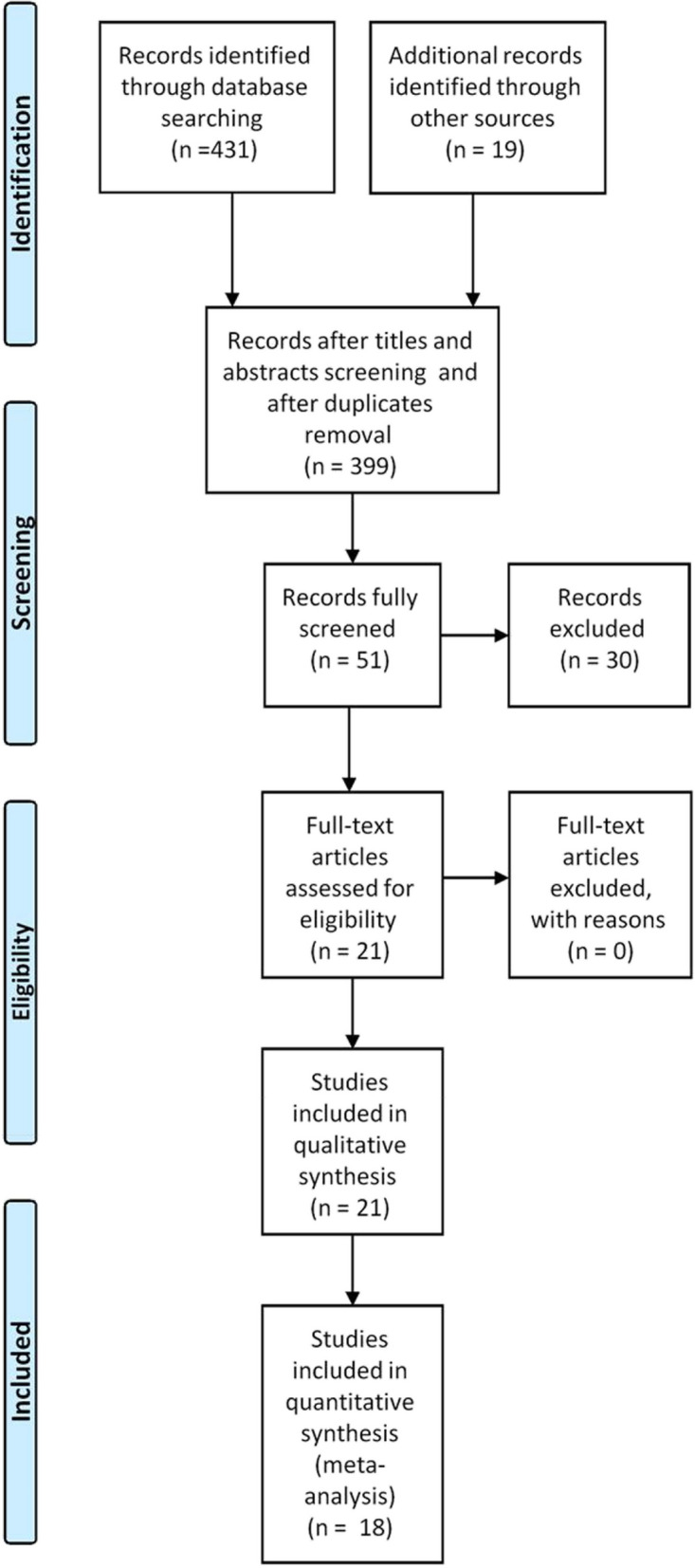
PRISMA flow diagram

A total of 450 articles were identified from the search strategy. From these, 399 articles were excluded. After abstract as well as full text screening twenty-one articles met the inclusion criteria and passed the quality assessment protocol. The articles reported prevalence among specific population and/or risk factors. (Fig. 1) illustrates the PRISMA diagram. The included articles are available in (Table 1). Assessment of the quality of included studies is depicted in (Table S2).

### Study characteristics

Twenty-one research articles were recruited to the study. Among witch eighteen research articles determined prevalence of causative microorganisms of Diarrhea [12–32]. The oldest were published in 2013 while the recent was published in 2022. Eight research articles determining prevalence of causative microorganisms of Diarrhea were conducted in Khartoum State, five in Gezira State, two in White Nile State, one in Kordofan State while two studies were conducted in several States. Moreover, 15 articles were conducted among both genders, while the remaining three studies did not determine the gender of their participants. Furthermore, majority of studies were concerned of prevalence among children while two studies were toward general population as well as mothers of children. All characteristics of included studies are depicted in (Table 1). Publication bias assessment indicated no major asymmetry.

### Prevalence of diarrhea

Prevalence estimates were synthesized to represent the overall burden as well as to estimate subgroup burden related to causative agents, study population and geographic location –whenever possible as illustrated below, summary of prevalence estimates synthesized from Diarrhea related included studies is available in (Table 2).

**Table 2:** Summary of prevalence estimates synthesized from diarrhea related included studies

Causative agent	Assessed in (State)	Assessed among	Total sample size	References	Pooled Prevalence	[95% CI]
Prevalence of viral Diarrhea	Khartoum, Gezira, Gadarif, Northern, South Darfur, North Kordofan & Red Sea	suspected children	14,132	(13,14,16,20,22,23,25,29,31–33)	22.90%	[15.37, 30.43]
Prevalence of *Rotaviral* Diarhhea	Khartoum, Gezira, Gadarif, Northern, South Darfur, North Kordofan & Red Sea	suspected children	12,758	(13,16,20,22,25,32,33)	22.21%	[9.97, 34.46]
Prevalence of parasite causing Diarhhea	Khartoum state, White Nile state & North Kordofan	suspected children, primary school adolescents & general population	1,100	(15,17,18,28,32)	31.40%	[19.53, 43.27]
Prevalence of *Hymenolepis nana*	Khartoum, Gezira & North Kordofan	Suspected children & adolescents	634	(15,21,28)	44.83%	[-11.57, 101.24]
Prevalence of *Giardia lamblia*	Khartoum, Gezira, North Kordofan & White Nile	General population, suspected children & adolescents	1,350	(15,18,21,28,32)	13.86%	[4.73, 22.99]
Prevalence of *Entameba.histolytica*	Khartoum, Gezira, North Kordofan and White Nile	General population, suspected children & adolescents	1,350	(15,18,21,28,32)	8.82%	[5.08, 12.56]
Prevalence of bacterial Diarhhea	Khartoum	Suspected children & adults	1,774	(16,19,32)	36.20%	[14.00, 58.40]
Prevalence of *Escherichia Coli*	Khartoum	Suspected children & adults	1,774	(16,19,32)	23.79%	[6.17, 41.41]
Prevalence of *Shigella spp.*	Khartoum	Suspected children & adults	1,774	(16,19,32)	7.04%	[3.84, 10.24]
Prevalence of *Salmonella spp.*	Khartoum	Suspected children & adults	1,774	(16,19,32)	1.94%	[0.66, 3.21]

### Prevalence of viruses causing diarrhea among children

**Fig. 2:**
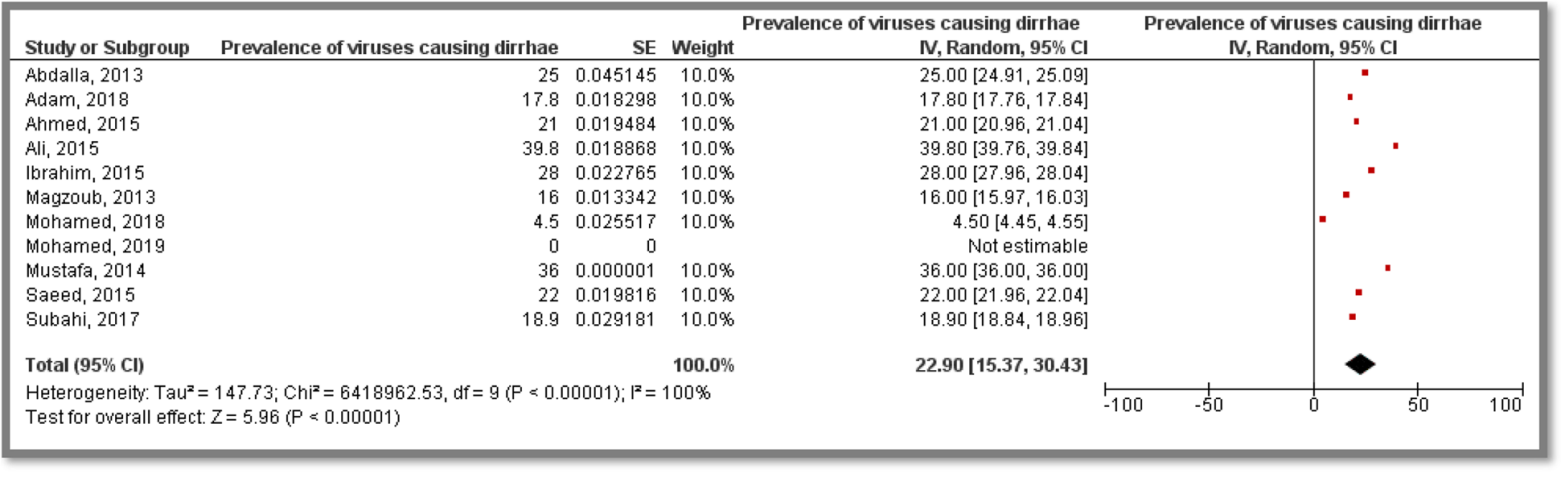
Meta analysis of prevalence of viral diarrhea among Sudanese children

Among the eighteen studies concerned with prevalence; eleven included studies provided viruses prevalence among participants, five studies were conducted in Khartoum State, four in Gezira State and two studies were conducted in different States representing a total sample size of 14,132 participants. The studies investigated prevalence of *Rotavirus*, *Adenovirus*, *Astrovirus* and *Bocavirus*. All studies targeted suspected children less than five years old from both gender. Only one study did not determine the gender of their participants. The pooled prevalence of viral diarrhea was 22.90% [15.37, 30.43]. Heterogeneity was high (I^2^ = 100%) (Fig. 2).

### Prevalence of rotavirus causing diarrhea among children

**Fig. 3:**
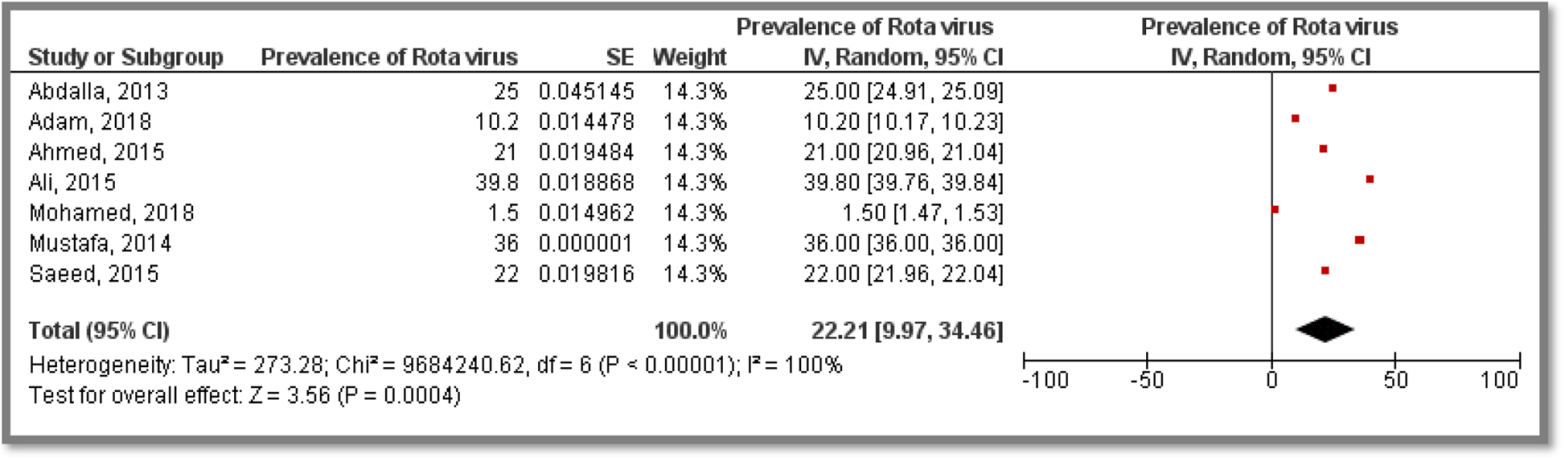
Meta analysis of prevalence of *Rotavirus* causing diarrhea among Sudanese children

Six included studies provided *Rotavirus* prevalence among participants; studies were conducted in Khartoum, Gezira as well as different States representing a total sample size of 12,758 participants concerning suspected children from both gender. The pooled prevalence was 22.21% [9.97, 34.46]. Heterogeneity was high (I^2^ = 100%) (Fig. 3).

### Prevalence of parasites causing diarrhea

**Fig. 4:**
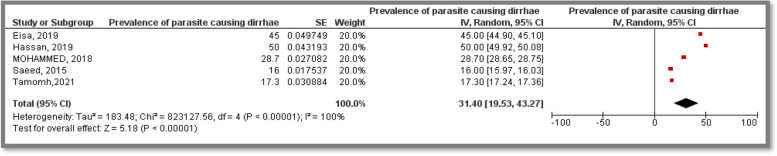
Meta analysis of prevalence of parasite causing diarrhea among Sudanese population

Five included studies provided parasites prevalence among participants, among which two studies were conducted in each of Khartoum State, as well as White Nile State while the last study participants were from Kordofan State representing a total sample size of 1,100 participants. The related articles covered *E.Histolytica, G.Lambilia, G.Intestinalis, H.Nana, E.vermicularis, A.Lumbricoids, S.Mansonia, Teania spp, S.Stercoralis* prevalence. Three studies concerned with children and another study was toward primary school adolescents of up to fourteen years old while one study was conducted among general population of all ages. The pooled prevalence was 31.40% [19.53, 43.27]. Heterogeneity was high (I^2^ = 100%) (Fig. 4).

### Prevalence of Hymenolepis nana causing diarrhea

Three included studies provided *Hymenolepis nana* prevalence among participants, the related studies were conducted in Khartoum, Gezira and Kordofan States representing a total sample size of 634 participants including suspected children as well as children and adolescents of up to 16 years old from both gender. The pooled prevalence was 44.83% [-11.57, 101.24]. Heterogeneity was high (I^2^ = 100%).

### Prevalence of Giardia and Entamoeba histolytica causing diarrhea

**Fig. 5:**
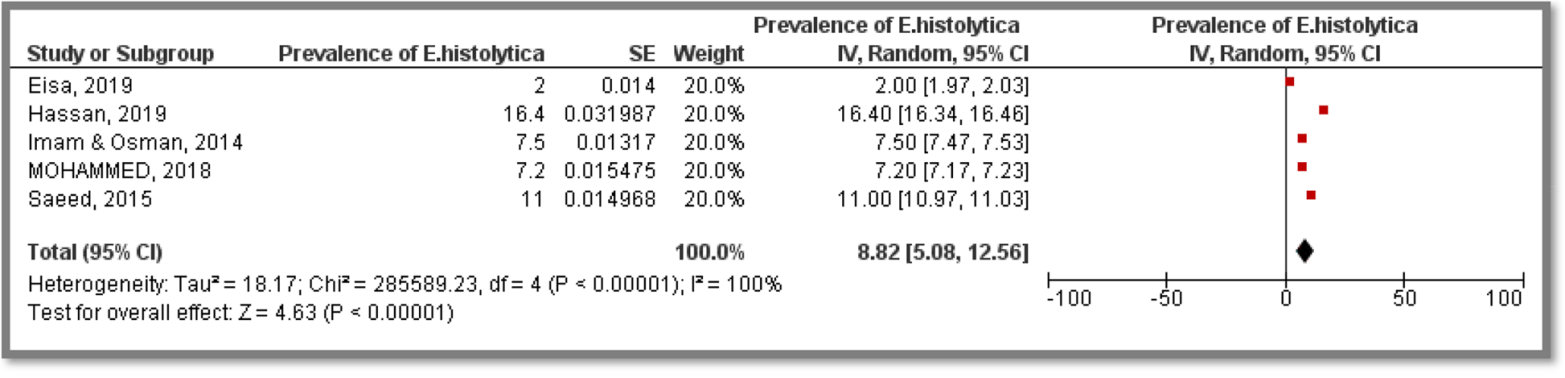
Meta analysis of prevalence of *E. histolytica* causing diarrhea among Sudanese participants

**Fig. 6:**
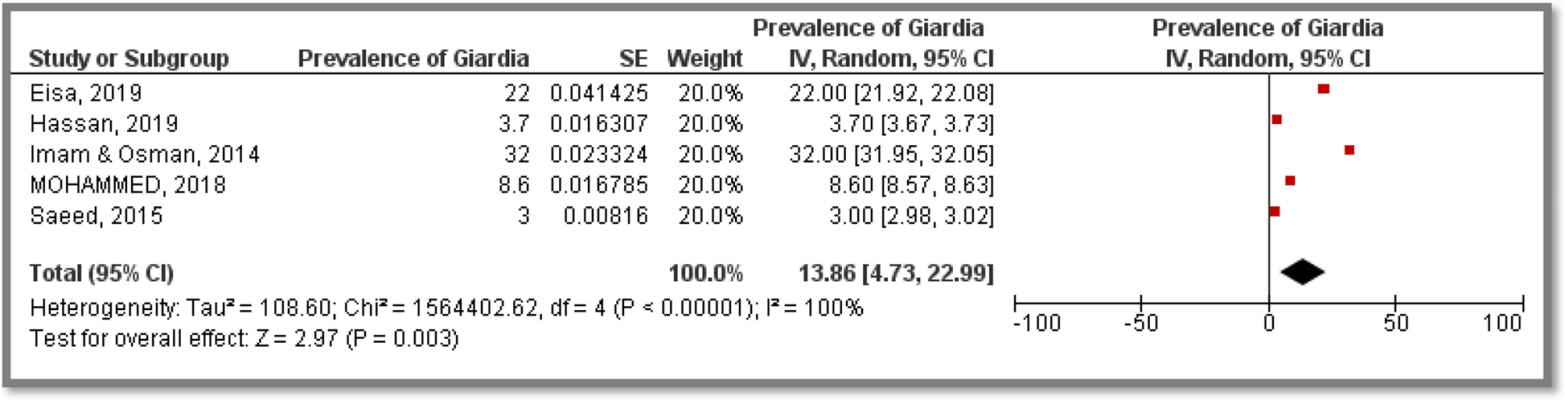
Meta analysis of prevalence of *Gardia* causing diarrhea among Sudanese participants

Five included studies provided *Giardia* as well as *E. histolytica* prevalence estimates among participants. The related studies were conducted in Khartoum, Gezira, Kordofan and White Nile States representing a total sample size of 1,350 participants. The majority of included research articles targeted suspected children as well as children and adolescents of up to 16 years old from both genders. Only one study was conducted among general population of all ages did not determine the gender of their participants. The pooled prevalence estimates were 13.86% [CI: 4.73, 22.99] and 8.82% [CI: 5.08, 12.56] for *Giardia* and *E. histolytica*, respectively. Heterogeneity was high in both Meta analysis (I^2^ = 100%) (Figs. 5 and 6).

### Prevalence of bacteria causing diarrhea

Three included studies investigated bacteria causing Diarrhea prevalence among participants; all studies were conducted in Khartoum State representing a total sample size of 1,774 participants. Studies investigated prevalence estimates of *Escherichia Coli, Shigella spp, Salmonella spp, Campylobacter spp, Aeromohydrophila, Morganella Morgani, Y. Enterocolitica and Non-Cholera vibrio*. Two studies were conducted among suspected children from both genders while the third study was carried out among children and adults without gender determination. The pooled prevalence was 36.20% [14.00, 58.40]. Heterogeneity was high (I^2^ = 100%).

### Prevalence of Escherichia Coli, Shigella spp and Salmonella spp causing diarrhea

Three included studies provided *E.Coli, Shigellaspp and Salmonella spp* prevalence estimates among participants. All studies were conducted in Khartoum State representing a total sample size of 1,774 participants. Two studies were conducted among suspected children from both genders while the third study was carried out among children and adults without gender determination. The pooled prevalence estimates were 23.52% [CI: 10.12, 36.93], 7.04% [CI: 3.84, 10.24] and 1.94% [CI: 0.66, 3.21] for *E. Coli, Shigella spp and Salmonella spp*, respectively. Heterogeneity was high in all Meta analysis (I^2^ = 100%).

### Socio-cultural risk factors of diarrhea

#### Education

Parent’s education was investigated as a possible socio-cultural risk factor toward children Diarrhea in 2 included studies among participants from different States. Among which; 6378 parents were classified as illiterates, the pooled odd ratio of their children having diarrhea was 5.34 [0.17, 167.93] with insignificant *p* value z = 0.95 (*P* = 0.34).

## Discussion

Prevalence of viral Diarrhea among children was investigated in seven States in Sudan, representing a total sample size of 14,132 participants recruited in eleven included studies; the overall pooled prevalence was 22.90% [CI:15.37, 30.43]. Moreover, prevalence of Rotaviral Diarhhea among children was investigated in seven States in Sudan, representing a total sample size of 12,758 participants recruited in seven included studies, the overall pooled prevalence was 22.21% [CI:9.97, 34.46]. This high prevalence may be due to the low level of sanitation reported previously in East African countries [34]. The pooled prevalence of diarrhea diseases among children generated from this study is considered high when compared to a study done in India (18%) [35], Egypt (19.5%) [36] and Ghana (19.2%) [37]. However, this finding is low compared to estimates concluded in India (25.2%) [38] as well as Ethiopia (23.1%) [39], this dissimilarity is possibly attributable to a difference in socio-demographic characteristics, climate, culture of stool disposal, water access, and the attitude of hand washing.

Prevalence of parasite causing Diarhhea was investigated in three States in Sudan representing a total sample size of 1,100 participants in five included studies; the overall pooled prevalence was 31.40% [CI:19.53, 43.27]. Moreover, prevalence of *Hymenolepis nana* was investigated in three States in Sudan representing a total sample size of 634 participants in three included studies; the overall pooled prevalence was 44.83% [CI:-11.57, 101.24]. Lower prevalence of protozoa was reported as (21.4%) [95% CI: 17.0 – 26.6] among children of northern Mozambique [40]. Moreover, studies from Nepal and Ethiopia indicated lower estimates as well (0.7%, 15.6%, respectively) [41, 42]. Nevertheless, in a recent study titled “Molecular prevalence of intestinal parasites infections in children with diarrhea in Franceville, Southeast of Gabon “ prevalence of pediatric diarrhea was concluded to be (61%) and that *Hymenolepis* species was the most common pathogen accounting for 31% of cases. Moreover, similar findings were reported from Burkina-Faso as well [43], Tanzania and South Africa reported higher estimates (55.6%, 68%, respectively) [44, 45]. However, the fact that some of these studies used molecular diagnostics with higher sensitivity is to be considered.

Prevalence of *Giardia lamblia *was investigated in four States in Sudan representing a total sample size of 1,350 participants in five included studies; the overall pooled prevalence was 13.86% [CI:4.73, 22.99]. Moreover, prevalence of *Entameba. histolytica* was investigated in four States in Sudan representing a total sample size of 1,350 participants in five included studies; the overall pooled prevalence was 8.82% [CI:5.08, 12.56]. However, lower as well as higher estimates have been reported for *G. lamblia* as (9.7%) and (17.2%) in several studies while lower *E. histolytica* as (0.4%) and (1%) were reported as well [40, 46, 47].

Prevalence of bacterial Diarhhea was investigated in Khartoum State representing a total sample size of 1,774 participants in three included studies; the overall pooled prevalence was 36.20%[CI:14.00, 58.40]. This finding is considered comparable to some extent of findings in United States as bacterial diarrhea was estimated to be approximately 31% of all diarrheas [48]. Moreover, prevalence of *Escherichia Coli* was investigated in Khartoum State representing a total sample size of 1,774 participants in three included studies; the overall pooled prevalence was 23.79%[CI:6.17, 41.41]. Furthermore, prevalence of *Shigella* spp. was investigated in Khartoum State representing a total sample size of 1,774 participants in three included studies; the overall pooled prevalence was 7.04% [CI:3.84, 10.24], while prevalence of *Salmonella* spp. was investigated in Khartoum State representing a total sample size of 1,774 participants in three included studies; the overall pooled prevalence was 1.94% [CI:0.66, 3.21]. Global estimates for the prevalence of *E.coli* causing diarrhea was reported as 10% to 25% which is in accordance to the findings of the current study. Moreover, pooled prevalence synthesized regarding *Shigella* spp as well as *salmonella* spp causing diarrhea is lower than estimates reported from Argentina and Djibouti as 10% and 3% for *Shigella* spp and *Salmonella* spp, respectively [49, 50].

Determining diarrheal causative pathogens to the strain level is crucial in understanding specific patterns of geographically related strain distributions [51], no study among included in the current review was found adopting typing of specific strains to better investigate the epidemiological burden of specific genotype causing diarrhea among children in Sudan.

The strengths of this review are that we systematically identified and included prevalence estimates, all studies with good quality were enrolled. Moreover, we have conducted meta-analysis to derive a pooled prevalence estimate of all included prevalence studies. Furthermore, we carried out a quality assessment of the included studies based on criteria specifically developed to determine the quality and the degree of selection bias in the studied populations.

However, several limitations are to be considered when interpreting study findings. First, gray literature evidence was not fully assessed, although all included studies are of good quality, several good studies might have been missed. Furthermore, the included studies used in the current study to determine pooled prevalence estimates adopted different approaches toward prevalence determination as molecular detection techniques [52] are not used for diagnosis purposes in Sudan. Lastly, the heterogeneity was high among the meta-analysis conducted and as consequence of the low sample size; sensitivity analysis was not able to be synthesized.

## Conclusion

The pooled prevalence of viral diarrhea was 22.90% among more than 14 thousands participants; the pooled prevalence of parasitic diarrhea was 31.40% while the pooled prevalence of bacterial diarrhea was 36.20%. No associated risk factors were able to be synthesized from included studies. Further research with larger sample sizes targeting prevalence, risk factors of diarrhea, molecular detection of virulence or specific pathological determinants among Sudanese children is needed to be conducted.

### Supplementary Information

**Additional file 1: Table S1.** PRISMA checklist of included studies. **Table S2.** Quality assessment of included studies.

## Data Availability

All related data are available in the manuscript.
